# Clinical perspectives on neuropsychological rehabilitation: challenges, expectations, and family involvement

**DOI:** 10.3389/fnhum.2025.1581304

**Published:** 2025-07-17

**Authors:** Carlos Ramos-Galarza, Jhonny Gaibor

**Affiliations:** ^1^Facultad de Salud y Bienestar, Carrera de Psicología, Pontificia Universidad Católica del Ecuador, Quito, Ecuador; ^2^Attention Area for Patients with Acquired Brain Injury, Hospital Eugenio Espejo, Quito, Ecuador

**Keywords:** cognitive rehabilitation, acquired brain damage, neuropsychology, traumatic brain injury, neuropsychological rehabilitation

## 1 Introduction

Neuropsychological rehabilitation (NR) is a comprehensive clinical approach aimed at restoring and compensating for cognitive, emotional, behavioral, and psychosocial impairments resulting from acquired brain injury (ABI) or neurological conditions (Cicerone et al., 2019; Wilson, 2014). Unlike purely cognitive rehabilitation, which focuses on specific domains such as memory or attention, NR encompasses a broader, interdisciplinary strategy designed to facilitate real-life functional recovery and reintegration into social roles (Prigatano et al., 1984).

The origins of NR can be traced back to early interventions with soldiers during World War I, where clinicians first recognized the complex consequences of brain injuries beyond physical deficits (Goldstein, 1942; Luria, 1980; Wilson, 2008). Over time, NR has evolved to incorporate advances in neuroscience, psychology, and clinical care, grounded in principles such as neuroplasticity (Cramer et al., 2011). Today, NR is used to support patients with a variety of neurological conditions, including traumatic brain injury, stroke, tumors, epilepsy, infections, and neurodevelopmental disorders (Polanowska, 2020; Kotelnikova et al., 2024).

In this opinion article, we aim to share reflections grounded in our clinical experience on key factors that influence the effectiveness of neuropsychological rehabilitation. We discuss the need for individualized treatment protocols, the emotional and practical involvement of families, the role of patient awareness and expectations, and the challenges associated with treatment dropout. Our goal is to provide clinical insights that can inform more effective rehabilitation strategies and improve long-term outcomes for individuals with ABI.

## 2 Factors influencing cognitive rehabilitation for brain-damaged patients

### 2.1 Why not follow the same neuropsychological rehabilitation process with all patients?

NR cannot be standardized across all patients with ABI because each individual presents with unique neurological, psychological, and social profiles. Several essential factors underscore the need for individualized treatment planning:

(a) Diversity of injuries and clinical presentations: ABI can result from diverse etiologies such as traumatic brain injury, stroke, or encephalitis. These vary in lesion location, size, and severity, which directly influence the patient's cognitive and behavioral profiles (Wilson, 2008). For instance, a left hemisphere stroke may result in aphasia, while a frontal lobe injury could cause executive dysfunction and emotional dysregulation.

(b) Neuroplasticity: One of the key principles guiding NR is neuroplasticity—the brain's capacity to reorganize and establish new connections following injury. This plasticity is most active during early development, yet persists to a lesser extent across the lifespan (Cramer et al., 2011). Younger patients tend to exhibit better recovery potential due to higher plasticity levels, making restorative techniques more effective. In contrast, older individuals may benefit more from compensatory strategies that promote functional adaptation rather than restoration.

(c) Psychological and emotional differences: Emotional responses to injury, including grief, denial, depression, or anxiety, vary significantly between individuals. Some patients may experience heightened emotional distress, while others display resilience. These psychological factors influence treatment engagement and outcomes (Kreutzer et al., 2009).

(d) Personal goals and sociocultural context: A patient's rehabilitation should align with their real-life objectives, such as returning to work, driving, or maintaining interpersonal relationships. Cultural background, family dynamics, and access to resources also affect how goals are set and pursued (Oshomoji et al., 2025; Prigatano, 2024).

(e) Interdisciplinary collaboration: Effective NR is typically delivered through a multidisciplinary team, including neuropsychologists, occupational therapists, speech-language pathologists, social workers, and neurologists. Coordination among professionals is necessary to address all functional domains and adapt to evolving needs over time (Ben-Yishay and Diller, 2011; Cicerone et al., 2019).

(f) Level of self-awareness: Perhaps one of the most critical, yet often underestimated, elements of individualized NR is the patient's level of self-awareness. Many ABI patients present anosognosia, a neurological deficit in which the individual lacks insight into their impairments (Zakrzewski and Rosen, 2014; Vuilleumier, 2004). This condition can significantly hinder engagement and adherence to treatment. Patients with intact self-awareness are generally more likely to participate actively and benefit from goal-directed interventions (Krivošíková and Angerová, 2021).

### 2.2 Family reactions and adaptation following an acquired brain injury diagnosis

In the acute and subacute phases of NR, it is common to observe intense emotional reactions from families upon receiving an ABI diagnosis. This emotional impact is often shaped by the sudden and disruptive nature of the injury, leading to grief, denial, shock, and even resistance to accepting the diagnosis, especially when it involves long-term or permanent cognitive and behavioral consequences (Allen et al., 2022).

Families frequently hold onto the hope of full recovery, which can generate unrealistic expectations and an unwillingness to acknowledge the severity or chronicity of the patient's deficits. In some cases, this may lead to the pursuit of unproven interventions or “miracle cures,” fueled more by emotional desperation than scientific evidence. Such pathways can divert families away from structured, evidence-based care, potentially delaying therapeutic engagement at a critical period in the patient's recovery (Al-Adawi et al., 2012).

On the other hand, families who succeed in accepting the diagnosis often display greater emotional resilience and adaptability. These families are more likely to actively participate in the therapeutic process and form a constructive alliance with the clinical team. Their ability to reorganize family roles, accept new caregiving responsibilities, and integrate psychoeducational guidance is associated with improved rehabilitation outcomes (Backhaus et al., 2021).

However, adaptation is not linear. Many families experience crises when faced with the reality of persistent impairments. Emotional overload, uncertainty about the future, and pre-existing family conflicts can amplify stress. These situations often require structured psychological support, especially in the form of family counseling, support groups, and educational interventions tailored to their needs and level of understanding (Braine, 2011).

The role of psychoeducation is central in this phase. Well-structured psychoeducational programs have been shown to help families understand the nature of ABI, interpret patient behaviors accurately, and develop realistic expectations about recovery. These programs also empower families to become active participants in the rehabilitation journey, enhancing their emotional preparedness and reducing the risk of burnout (De Luca et al., 2022).

### 2.3 The role of the patient and the challenges of neuropsychological rehabilitation

The degree to which a patient with ABI can engage in NR is strongly influenced by a combination of neurological, psychological, and contextual variables. These include the type, severity, and location of the brain injury, as well as the patient's baseline personality traits, emotional resilience, self-awareness, and support systems (Gutbrod et al., 2017).

Neurological severity, often assessed using tools such as the Glasgow Coma Scale, post-traumatic amnesia duration, or neuroimaging, can predict a patient's initial level of awareness, attention, and capacity for active participation in therapeutic tasks. Patients with severe injuries may initially require a passive role, relying heavily on structured, externally guided interventions. In contrast, patients with mild to moderate injuries may assume a more collaborative and autonomous role, actively engaging in goal setting and self-monitoring during rehabilitation (Kwak et al., 2020).

Furthermore, the psychosocial impact of the injury must not be underestimated. ABI often results in significant changes to the patient's roles and identity, including loss of employment, dependency in daily activities, and strained interpersonal relationships. These disruptions can contribute to depression, demotivation, and withdrawal, which are known to interfere with rehabilitation outcomes. Psychotherapeutic support, motivational interviewing, and cognitive-behavioral strategies are essential to address these issues and foster emotional readiness (Carnes and Quinn, 2005).

The context of rehabilitation, including access to services, socio-economic conditions, and family support, also plays a vital role. Patients from underserved backgrounds may face logistical and financial barriers that reduce continuity of care. Likewise, cultural factors may influence the patient's understanding of their condition and expectations for recovery. A culturally responsive and context-sensitive approach to NR is essential to overcome these systemic challenges (Oshomoji et al., 2025).

### 2.4 Expectations and misconceptions in neuropsychological rehabilitation

In patients and families confronting the aftermath of ABI, expectations regarding the rehabilitation process often oscillate between overconfidence and despair. While some maintain an optimistic view of complete recovery, others perceive minimal improvement as evidence of therapeutic failure. These unrealistic or binary expectations can significantly impair motivation, adherence, and emotional resilience (Stejskal, 2012).

Research shows that families frequently hope for a full return to premorbid functioning, particularly in cases of mild to moderate injuries where physical recovery progresses faster than cognitive or emotional recovery. This mismatch between visible recovery and invisible deficits, such as executive dysfunction or memory problems, often leads to frustration and disappointment when patients do not meet initial timelines or expected milestones (Blanton et al., 2018).

Cultural beliefs, misinformation, and lack of education may also shape expectations. Some families resort to non-evidence-based treatments or delay formal rehabilitation, expecting spontaneous recovery or “miraculous” improvements through prayer, rituals, or unverified therapies. Although culturally rooted hope can be emotionally supportive, excessive reliance on magical thinking may result in neglecting time-sensitive and critical early neurorehabilitation strategies that are crucial for brain plasticity.

These dynamics underscore the need for structured psychoeducation, a central intervention that helps align expectations with the scientific reality of neuropsychological recovery. Well-designed psychoeducational programs facilitate understanding of the brain's healing trajectory, the complexity of functional outcomes, and the importance of consistency and engagement over time (Ekhtiari et al., 2017).

Psychoeducation should not only focus on patients, but also involve their family and caregivers as active stakeholders. Clinicians must adopt a communicative style that is transparent, empathetic, and tailored to the individual's educational level and emotional state. Through this approach, unrealistic expectations can be replaced by informed hope, where patients and families recognize the challenges ahead while remaining engaged in the rehabilitation journey.

### 2.5 The essential role of family participation in neuropsychological rehabilitation

Family participation is a cornerstone of successful NR, particularly for individuals recovering from ABI. The family's involvement extends beyond emotional support, significantly influencing adherence to treatment, functional recovery, and long-term psychosocial outcomes.

Families serve as co-therapists, reinforcing therapeutic techniques at home, facilitating daily routines, and helping patients apply learned strategies to real-world contexts. Their participation ensures that rehabilitation extends beyond the clinical environment, promoting consistency, motivation, and generalization of skills (Kreutzer et al., 2009).

However, this involvement is often accompanied by psychological strain, particularly when families must suddenly assume caregiving roles without prior preparation. Common challenges include emotional overload, financial stress, social isolation, and role conflicts, especially when the injured person had been a primary income earner or caregiver themselves (Verhaeghe et al., 2005).

The quality of family functioning, including communication patterns, cohesion, and conflict resolution skills, also affects the rehabilitation process. Families with pre-existing conflict may experience increased tension and reduced caregiving efficacy, while well-functioning families can buffer the patient from psychological distress and environmental instability (Pitschel-Walz et al., 2011).

As such, the integration of family-centered interventions into NR is vital. These include:

- Psychoeducation, to enhance understanding of the injury, prognosis, and treatment process.
- Skills training, including communication, problem-solving, and stress management techniques.
- Psychological support, such as counseling or support groups, to reduce caregiver burden and prevent burnout.

Furthermore, family participation is often dynamic, beginning with high motivation and involvement, but later declining due to cumulative fatigue, disillusionment, or logistical barriers. Clinicians must monitor family engagement longitudinally and provide booster sessions, flexible communication, and tailored support plans to maintain commitment over time.

### 2.6 Patient attrition in neuropsychological rehabilitation: barriers and solutions

One of the most significant challenges in NR is patient attrition (the premature discontinuation of treatment despite ongoing clinical need). Attrition is often driven by a complex interaction of cognitive, emotional, socioeconomic, and systemic factors, and its consequences are serious: reduced functional recovery, increased caregiver burden, and higher long-term healthcare costs (Singh and Kaloiya, 2020).

From a cognitive standpoint, conditions like anosognosia or executive dysfunction can impair the patient's ability to recognize their deficits, organize their schedule, or follow through with therapeutic activities (Prigatano et al., 2023). These patients may believe that they are fully recovered or that rehabilitation is unnecessary, which contributes to poor engagement and eventual dropout (Steward and Kretzmer, 2021).

Emotionally, patients with ABI frequently face depression, frustration, shame, or loss of identity, factors that diminish motivation, hope, and treatment adherence. When expectations for recovery are overly optimistic and not met quickly, disappointment can lead to withdrawal from therapy (Coetzer, 2009).

Systemic and environmental issues compound these personal struggles. Logistical barriers, such as transportation difficulties, work or family obligations, or the lack of flexible scheduling, are common causes of missed sessions. In low- and middle-income contexts, financial limitations and poor access to follow-up care frequently result in early dropout, especially among underserved populations (Legg et al., 2022).

Addressing attrition requires a multi-level strategy. Early psychoeducation is crucial for aligning patient and family expectations, enhancing insight, and clarifying the non-linear and prolonged nature of recovery. Motivational interviewing, metacognitive interventions, and emotion-focused therapies can help patients reconnect with their sense of agency and purpose. Moreover, integrating caregiver support into treatment reduces the burden and fosters sustained participation (Bivona et al., 2022).

On a structural level, telehealth and home-based NR programs have shown promising results in reducing dropout, especially for patients with transportation or mobility issues. Rehabilitation programs must also adopt flexible, culturally sensitive, and person-centered models that accommodate the specific needs and values of each patient (Appleman et al., 2021).

## 3 Discussion

NR is a multifaceted process that involves more than restoring cognitive functions; it demands a deep understanding of the patient's neurological condition, psychological state, social context, and family dynamics. In this opinion article, we have discussed six critical dimensions that regularly emerge in clinical settings when treating patients with ABI: the need for individualized rehabilitation, family reactions to diagnosis, patient participation and insight, the role of expectations, the impact of family engagement, and the phenomenon of treatment attrition.

First, we emphasized the need to reject one-size-fits-all approaches. Given the diversity of ABI presentations and recovery potential, tailoring rehabilitation plans to individual profiles is fundamental. Neuroplasticity provides a scientific foundation for optimism, but its application must be realistically calibrated based on age, severity, and co-occurring factors.

Second, the emotional and behavioral responses of families to diagnosis profoundly shape the therapeutic environment. When families move beyond denial and engage with structured psychoeducation and support, they become not just caregivers but therapeutic allies. Conversely, unresolved grief or unrealistic expectations can become obstacles to rehabilitation progress.

Third, the role of the patient is heavily influenced by the severity of the injury, cognitive awareness, and emotional adjustment. Impaired self-awareness is one of the most challenging barriers to overcome, yet targeted strategies, such as metacognitive training and insight-oriented therapy, can improve engagement and long-term adherence.

Fourth, unrealistic expectations often hinder participation, especially when cultural or emotional narratives clash with the scientific realities of slow, non-linear recovery. Early and clear psychoeducation can prevent disillusionment and instead cultivate a mindset of “informed hope,” which is essential to sustaining therapeutic effort.

Fifth, active family participation enhances adherence, facilitates the transfer of learned strategies to daily life, and mitigates the emotional impact of disability. However, such involvement must be supported by institutional interventions that address emotional fatigue, role strain, and systemic barriers to continued caregiving.

Sixth, patient attrition remains a persistent issue in NR. It is rarely due to “lack of will,” but more often reflects cognitive, emotional, and structural barriers. Understanding these elements enables clinicians to proactively implement strategies such as telehealth, caregiver reinforcement, flexible scheduling, and motivational techniques to sustain engagement.

As we have seen through clinical observation and supported by scientific evidence, the success of NR depends not only on the therapeutic content, but also on the patient's ecosystem, their emotional resources, insight, family support, and the cultural and institutional context in which rehabilitation occurs.

Although this article presents reflections based on daily clinical practice, it also aims to encourage a broader dialogue about neglected or underestimated variables in rehabilitation planning. In future works, we intend to explore topics such as cultural interpretations of cognitive disorders, spiritual coping strategies, and neuroeducational counseling as tools for family empowerment and therapeutic alliance-building.

In conclusion, NR is both a scientific endeavor and a profoundly human one. It demands technical skill, cultural sensitivity, and relational commitment. We hope this contribution supports clinicians and researchers in designing interventions that are not only effective but compassionate and realistic.

## References

[B1] Al-Adawi S. Al-Busaidi Z. Al-Adawi S. Burke D. (2012). Families coping with disability due to brain injury in Oman: attribution to belief in spirit infestation and Ensorcellment. SAGE Open 2, 1–8. 10.1177/2158244012457400

[B2] Allen N. Hevey D. Carton S. O'Keeffe F. (2022). Life is about “constant evolution”: the experience of living with an acquired brain injury in individuals who report higher or lower posttraumatic growth. Disabil. Rehabil. 44, 3479–3492. 10.1080/09638288.2020.1867654 33459069

[B3] Appleman E. O'connor M. Boucher S. Rostami R. Sullivan S. Migliorini R.. (2021). Teleneuropsychology clinic development and patient satisfaction. Clin. Neuropsychol. 35, 819–837. 10.1080/13854046.2020.1871515 33504268

[B4] Backhaus S. L. Bergquist T. F. Ibarra S. L. Kreutzer J. S. (2021). “Practical approaches to family assessment and intervention,” in Brain Injury Medicine, eds. N. D. Zasler, D. I. Katz, R. D. Zafonte, D. B. Arciniegas, M. R. Bullock, F. M. Hammond, J. S. Kreutzer, R. Nakase-Richardson, and T. K. Watanabe (Springer Publishing Company), 1252–1267. 10.1891/9780826143051.008211261958

[B5] Ben-Yishay Y. Diller L. (2011). Handbook of Holistic Neuropsychological Rehabilitation: Outpatient Rehabilitation of Traumatic Brain Injury. New York, NY: Oxford University Press.

[B6] Bivona U. Azicnuda E. Rapiti M. Silvestro D. (2022). “Psycho-educational intervention on caregivers within the rehabilitation process: from the post-acute to the homecoming phases,” in Diagnosis and Treatment of Traumatic Brain Injury, eds. R. Rajendram, V. Preedy, and C. Martin (Cambridge, MA: Academic Press), 531–541. 10.1016/B978-0-12-823347-4.00006-3

[B7] Blanton S. Dunbar S. Clark P. (2018). Content validity and satisfaction with a caregiver-integrated web-based rehabilitation intervention for persons with stroke. Top. Stroke Rehabil. 25, 168–173. 10.1080/10749357.2017.1419618 29334344 PMC5876148

[B8] Braine M. (2011). The experience of living with a family member with challenging behavior post acquired brain injury. J. Neurosci. Nurs. 43, 156–164. 10.1097/JNN.0b013e3182135bb2 21796033

[B9] Carnes S. Quinn W. (2005). Family adaptation to brain injury: coping and psychological distress. Fam. Syst. Health 23, 186–203. 10.1037/1091-7527.23.2.186

[B10] Cicerone K. Goldine Y. Ganci K. Rosenbaum A. Wethe J. Langenbhn D.. (2019). Evidence-based cognitive rehabilitation: systematic review of the literature from 2009 through 2014. Arch. Phys. Med. Rehabil. 100, 1515–1533. 10.1016/j.apmr.2019.02.011 30926291

[B11] Coetzer R. (2009). A clinical pathway including psychotherapy approaches for managing emotional difficulties after acquired brain injury. CNS Spectrums 14, 632–638. 10.1017/S1092852900023877 20173688

[B12] Cramer S. Sur M. Dobkin B. O'Brien C. Sanger T. Trojanowski J.. (2011). Harnessing neuroplasticity for clinical applications. Brain 134, 1591–1609. 10.1093/brain/awr039 21482550 PMC3102236

[B13] De Luca R. Rifici C. Pollicino P. Parisi S. Bonanno M. Torregrossa W.. (2022). Is the “family glass cabin” useful to safely allow inpatient–caregiver interaction in the COVID-19 era? A pilot study on severe acquired brain injury. J. Clin. Med. 11, 1–10. 10.3390/jcm11061623 35329947 PMC8950736

[B14] Ekhtiari H. Rezapour T. Aupperle R. Pualus M. (2017). Neuroscience-informed psychoeducation for addiction medicine: a neurocognitive perspective. Progr. Brain Res. 235, 239–264. 10.1016/bs.pbr.2017.08.013 29054291 PMC5771228

[B15] Goldstein K. (1942). Aftereffects of Brain Injuries in War: Their Evaluation and Treatment: The Application of Psychologic Methods in the Clinic. New York, NY: Grune and Stratton. 10.1037/13578-000

[B16] Gutbrod K. Heinemann D. Müri R. (2017). Neurorehabilitation of cognitive disorders. Therapeut. Umschau 74, 503–509. 10.1024/0040-5930/a000948 29583098

[B17] Kotelnikova A. Kukshina A. Turova E. (2024). Differentiated approach to cognitive rehabilitation of patients after stroke. Probl. Balneol. Physiother. Ther. Phys. Cult. 6, 5–11. 10.17116/kurort20241010615 39718952

[B18] Kreutzer J. Rapport L. Marwitz J. Harrison-Felix C. Hart T. Glenn M.. (2009). Caregivers' well-being after traumatic brain injury: a multicenter prospective investigation. Arch. Phys. Med. Rehabilit. 90, 939–946. 10.1016/j.apmr.2009.01.010 19480869

[B19] Krivošíková M. Angerová Y. (2021). Self-awareness and its evaluation in patients after acquired brain injury. Kontakt 23, 193–199. 10.32725/kont.2021.027

[B20] Kwak E. Wi S. Kim M. Pyo S. Shin Y. Oh K.. (2020). Factors affecting cognition and emotion in patients with traumatic brain injury. Neurorehabilitation 46, 369–379. 10.3233/NRE-192893 32310194 PMC7306897

[B21] Legg M. Foster M. Jones R. Kendall M. Fleming J. Nielsen M.. (2022). The impact of obstacles to health and rehabilitation services on functioning and disability: a prospective survey on the 12-months after discharge from specialist rehabilitation for acquired brain injury. Disabil. Rehabilit. 44, 5919–5929. 10.1080/09638288.2021.1952321 34270367

[B22] Luria A. (1980). The Working Brain. New York, NY: Basic Books Inc.

[B23] Oshomoji O. Ajiraba J. Semudamara S. Olayemi M. Adeoye S. Ugwu O.. (2025). Cultural influences on physiotherapy engagement and outcomes in Guillain-Barré syndrome rehabilitation: a systematic review. Discover Public Health 22, 302. 10.1186/s12982-025-00682-8

[B24] Pitschel-Walz G. Froböse T. Krämer S. Gsottschneider A. Bäuml J. Jahn T. (2011). Effectiveness of psychoeducational groups in subjective experience in schizophrenia. Zeitschrift Klinische Psychol. Psychother. 40, 186–197. 10.1026/1616-3443/a000101

[B25] Polanowska K. (2020). Neuropsychological rehabilitation of acquired, non-progressive cognitive-behavioral disorders in evidence-based clinical recommendations. Rehabilit. Medyczna 24, 31–39. 10.5604/01.3001.0014.4134

[B26] Prigatano G. (2024). Disturbances in higher order consciousness encountered in neuropsychological rehabilitation and assessment. J. Int. Neuropsychol. Soc. 30, 913–922. 10.1017/S1355617724000705 39676690

[B27] Prigatano G. Fordyce D. Zeiner H. J. R. Pepping M. Wood B. (1984). Neuropsychological rehabilitation after closed head injury in young adults. J. Neurol. Neurosurg. Psychiatry 47, 505–513. 10.1136/jnnp.47.5.505 6736983 PMC1027828

[B28] Prigatano G. Russell S. Meites T. (2023). Studying lack of awareness of cognitive decline in neurodegenerative diseases requires measures of both anosognosia and denial. Front. Aging Neurosci. 15:1325231. 10.3389/fnagi.2023.1325231 38259640 PMC10800930

[B29] Singh G. Kaloiya G. (2020). “Neuropsychological rehabilitation,” in Examining Biological Foundations of Human Behavior, ed. B. Vijaya (IGI Global), 226–242. 10.4018/978-1-7998-2860-0.ch013

[B30] Stejskal T. (2012). Removing barriers to rehabilitation: theory-based family intervention in community settings after brain injury. Neurorehabilitation 31, 75–83. 10.3233/NRE-2012-0776 22523015

[B31] Steward K. Kretzmer T. (2021). Anosognosia in moderate-to-severe traumatic brain injury: a review of prevalence, clinical correlates, and diversity considerations. Clin. Neuropsychol. 36, 2021–2040. 10.1080/13854046.2021.1967452 34429014

[B32] Verhaeghe S. Defloor T. Grypdonck M. H. F. (2005). Stress and coping among families of patients with traumatic brain injury: a review of the literature. *J. Clin. Nurs*. 14, 1004–1012. 10.1111/j.1365-2702.2005.01126.x 16102152

[B33] Vuilleumier P. (2004). Anosognosia: the neurology of beliefs and uncertainties. Cortex 40, 9–17. 10.1016/S0010-9452(08)70918-3 15070000

[B34] Wilson B. (2008). Neuropsychological rehabilitation. Annu. Rev. Clin. Psychol. 4, 141–162. 10.1146/annurev.clinpsy.4.022007.141212 17716045

[B35] Wilson B. (2014). Neuropsychological Rehabilitation. Cambridge Handbook of Psychology, Health and Medicine, 2nd Edn. Cambridge, UK: Cambridge University Press.

[B36] Zakrzewski J. Rosen H. (2014). “Anosognosia,” in Encyclopedia of the Neurological Sciences, eds. M. Minoff, and R. Daroff (Elsevier), 198–201.

